# Disseminated *Mycobacterium tuberculosis*: An Unusual Presentation with Associated Hemophagocytic Lymphohistiocytosis

**DOI:** 10.1155/2022/4669025

**Published:** 2022-01-27

**Authors:** Ahmed Arfa, Nivin Omar, Kulsum Bano, Natasha M. Savage

**Affiliations:** Department of Pathology, Medical College of Georgia at Augusta University, Augusta, Georgia

## Abstract

Hemophagocytic lymphohistiocytosis (HLH) is a rare, potentially fatal, and systemic hyperinflammatory syndrome with exacerbated and uncontrolled activation of histiocytes and lymphocytes against mature cells. Secondary HLH can occur in association with a myriad of underlying infections or malignancies. Our patient is a 38-year-old male prisoner with poorly controlled diabetes and no known other medical conditions. He was referred to our emergency department with three-week history of worsening malaise, weight loss, fever, bruising, and shortness of breath; imaging showed pneumomediastinum, lung nodule, and adrenal mass. Biopsy of the lung nodule revealed acid-fast bacilli. Furthermore, bone marrow biopsy showed foci of necrosis with associated acid-fast bacilli and hemophagocytosis highlighted by CD163 stain; consequently, secondary HLH was suggested. Hence, lab results were reviewed and found to satisfy five of the eight secondary HLH criteria. Moreover, ferritin was >10,000 ng/ml, which has been suggested to be highly suspicious for HLH. The patient was started on anti-MAC therapy. Unfortunately, the patient's status declined rapidly; he developed multi-organ failure and succumbed to disease. Later, his culture confirmed *Mycobacterium tuberculosis*. In conclusion, we presented a rare and challenging case of secondary HLH associated with disseminated *Mycobacterium tuberculosis*. A high index of suspicion is required for early diagnosis and treatment, and pathologists should be aware of *Mycobacterium tuberculosis*' association with secondary HLH.

## 1. Introduction

Hemophagocytic lymphohistiocytosis (HLH) is a systemic, hyperinflammatory syndrome with uncontrolled activation of histiocytes and lymphocytes against mature cells and their hematopoietic precursors within the reticuloendothelial system [1]. Additionally, there is cytokine overproduction, and consequently, hypercytokinemia (so called “cytokine storm”) can cause end-organ failure and death. It can be misdiagnosed as sepsis, among other entities, given significant clinical and laboratory overlap [2]. HLH is classified into two forms, primary or familial, which is due to a variety of genetic defects, and secondary or acquired, which is associated with a myriad of causes including infections, malignancies, and autoimmune diseases [3]. Although HLH secondary to infection is predominantly viral in origin, especially Epstein–Barr virus (EBV), bacteria such as mycobacteria, fungi, and protozoa can trigger secondary HLH as well [4]. *Mycobacterium tuberculosis* (MTB) has diverse clinical presentations and complications; thus, it can be a great challenge for clinicians to diagnose. It is rarely complicated by HLH [1, 5]. Furthermore, spontaneous pneumomediastinum is a rare presentation of MTB [6]. Despite this, our patient presented with spontaneous pneumomediastinum and developed secondary HLH. Tuberculosis-associated HLH (TB-HLH) has high mortality rate [1, 5], and a high index of suspicion is required by pathologists and clinicians alike to start prompt treatment with anti-tuberculous therapy (ATT) [7]. We report an unusual presentation of disseminated MTB associated with secondary HLH in a patient with uncontrolled diabetes mellitus.

## 2. Case Description

A 38-year-old incarcerated male patient with a medical history of newly diagnosed type 2 diabetes mellitus and tobacco abuse was transferred to our inpatient facility for further evaluation of spontaneous pneumomediastinum. Three weeks prior to presentation, the patient noted increasing fatigue, shortness of breath on exertion, weight loss, and rash. On day of hospital admission, he was found to have mild splenomegaly and petechial rash involving the abdominal wall and lower extremities, with no other pertinent positive findings on physical examination. He underwent computerized tomography (CT) scans of chest, abdomen, and pelvis at an outside facility, which revealed a right lower lobe lung nodule, left adrenal mass, and small pneumomediastinum.

**Figure 1:**
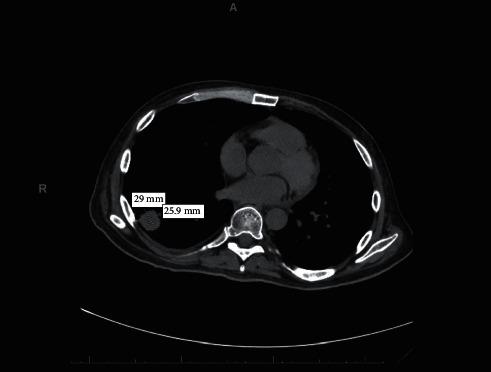
CT chest imaging showing increase in size of right lower lobe lung nodule.

Initial laboratory examination revealed normocytic anemia, hemoglobin of 8 g/dL, white blood cell (WBC) count of 9,300/mm^3^, platelet count of 76,000/mm^3^, absolute reticulocyte count of 1.7, fibrinogen of 321 mg/dL, prothrombin time /international normalized ratio (PT/INR) of 11.0 seconds/1.0, activated partial thromboplastin time (aPPT) of 26.3 seconds, lactate dehydrogenase (LDH) of 1138 U/L, haptoglobin <30 mg/dL, ferritin of 10,398 ng/mL, iron saturation of 68%, total iron binding capacity of 137 mcg/dL, total bilirubin of 0.7 mg/dL, aspartate aminotransferase (AST) of 66 U/L, alanine aminotransferase (ALT) of 57 U/L, and creatinine of 0.54 mg/dL. Bronchoscopy on hospital day 2 ruled out airway trauma as a cause for pneumomediastinum. Repeat CT imaging showed increase in size of right lower lobe lung nodule and stable size of left adrenal nodule (Figure 1).

**Figure 2:**
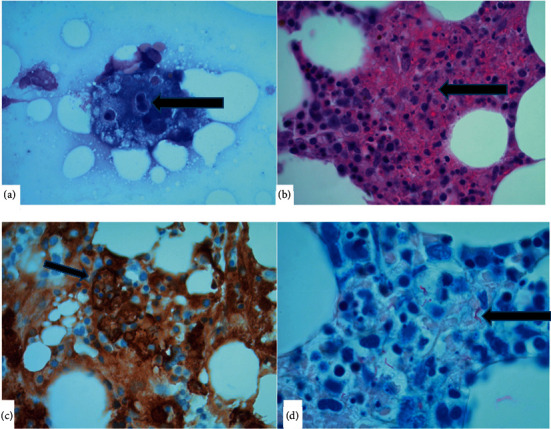
40x magnification. (a) Bone marrow aspirate smear showing histiocyte with hemophagocytosis. (b) Bone marrow biopsy H&E stain showing foci of necrosis by normal bone marrow. (c) CD163 stain showing histiocyte engulfing other hematopoietic cell (rosette appearance). (d) FITE stain showing acid-fast bacilli.

Differential diagnosis based on initial presentation included primary lung malignancy metastatic to adrenal gland versus malignancy of unknown primary versus infectious process. Differential diagnosis for cytopenia included anemia of chronic disease secondary to presumed malignancy, marrow infiltration secondary to metastatic disease or other marrow infiltrative process, and/or HLH related to malignancy or infection. On day 7 of admission, patient underwent CT guided biopsy of the right lower lobe lung nodule, which revealed numerous acid-fast bacilli (AFB), without evidence of malignancy. A bone marrow biopsy performed on day 14 revealed prominent increase in macrophages with hemophagocytosis within aspirate smears (Figure 2(a)). The bone marrow biopsy showed small areas of necrosis (Figure 2(b)). CD163 stain further highlighted the hemophagocytosis (Figure 2(c)). Acid-fast microorganisms were noted on FITE stain (Figure 2(d)). The H Score [8] was utilized, and a score of 195 was obtained, indicating an 80–88% probability of HLH. Hence, a preliminary diagnosis of disseminated mycobacterial infection with secondary HLH was made. Initial suspicion was for *Mycobacterium avium* complex (MAC), and the patient was initiated on empiric anti-MAC therapy with amikacin, rifampin, ethambutol, and azithromycin. Unfortunately, before HLH directed therapy could be initiated, the patient had clinical deterioration with multi-organ failure (acute liver and renal failure), disseminated intravascular coagulation, and acute hypoxic respiratory failure requiring mechanical ventilation. The patient's clinical condition continued to rapidly deteriorate, and he subsequently expired on day 18. Posthumously, we received results of AFB cultures from bronchoalveolar lavage indicating MTB infection.

## 3. Discussion

HLH is a life threating disorder first described in 1952 as “familial hemophagocytic reticulosis.” It is associated with hyperinflammatory syndrome and cytokine storm as a result of uncontrolled, exacerbated Th_1_-cell and macrophage activation with diminished ability of cytotoxic T-cells and natural killer (NK) cells clearing up the target antigen. This results in ineffective inflammatory response and uncontrolled histiocytic phagocytosis of blood trilinear precursors and the mature blood elements in bone marrow. There are two forms of the disease: primary form also referred to as familial HLH and secondary or acquired HLH [1]. The primary autosomal recessive form has an incidence rate of 1 : 50,000 live births. The median survival rate is less than two months after diagnosis if left untreated [9]. It typically presents during infancy or early childhood, and it may be triggered by infections [10]. Secondary HLH can occur secondary to many underlying diseases including lymphoma (anaplastic large cell lymphoma, NK/T-cell lymphoma nasal type, Hodgkin lymphoma, and so on), infection (EBV, cytomegalovirus, herpes simplex viruses, adenovirus, and so on), autoimmune disease (lupus, systemic idiopathic juvenile arthritis, and so on), post-allogeneic stem cell transplant (immunologic reaction at engraftment), or medication-related hypersensitivity (carbamazepine, phenobarbital, sulfamethoxazole, and so on).

In secondary HLH, it is important to identify the underlying cause as the prognosis varies greatly based at least in part by different underlying conditions. Although infection-related HLH is predominantly viral in origin, especially EBV, bacteria such as mycobacteria, fungi, and protozoa can trigger secondary HLH as well [4, 11]. TB-HLH was first reported in 1980s [8]. MTB is an obligate intracellular pathogen that induces TH1-mediated cytotoxicity with activation of NK-cells and macrophages resulting in the release of chemokines and cytokines including TNF-***α***, IFN-*γ*, IL-1, IL-6, IL-18, and GM-CSF, thus resulting in a cytokine storm [1]. In Per Tseng et. al's study 23% of all infection induced HLH was related to MTB. It was also associated with longer duration of symptoms and a higher mortality rate [5]. This was reflected in our patient's case, as our patient died as a result of TB-HLH.

Most TB-HLH patients present with a fever of unknown origin (FOU), hepatosplenomegaly, and cytopenias [12]. However, to the best of our knowledge, this is the first reported TB-HLH case to present with spontaneous pneumothorax. Our patient had multiple risk factors contributing to the development of TB including a history of type 2 diabetes and incarceration.

TB-HLH has a varied course, with an overall high mortality. Given the rarity of the condition and typical delays in diagnosis, patients with TB-associated HLH tend to have poorer outcomes, with reported mortality ranging from 50% [13] to 100% [1] without treatment. Padhi et al. reported that based on a literature review performed from January 1975 to March 2014, there was 63 cases of TB-HLH with a reported fatality rate of 49% (31 of the 63 patients studied) [1].

Bhattacharyya et al. reported that there may be a delay in diagnosis of HLH for multiple reasons including concordant liver failure and coagulopathy. In our case, our patient developed liver dysfunction and coagulopathy, which may have obscured/delayed the TB-HLH diagnosis [5]. Moreover, there is significant clinical overlap between HLH and septic shock, thus further complicating diagnosis; however, HLH can be diagnosed if there is a mutation in a known causative gene (primary/familial) or if at least five of eight HLH-2004 criteria are met (secondary) as shown in Table 1. Our patient fulfilled total five criteria out of eight as per HLH-2004 protocol including (cytopenias, fever, splenomegaly, hemophagocytosis, and hyperferritinemia). A new scoring system was introduced in 2014 called H Score which helps to avoid under-diagnosis of HLH. It consists of nine variables as shown in Table 2. There is a possible number of points assigned to each variable, and then the H Score is calculated. Our patient had a score of 195, indicating an 80–88% probability of HLH.

Regarding therapy, the goal of treatment in HLH is to curtail the widespread inflammation and multi-organ failure caused by excessive activation of the immune system [15]. This consists of a regimen of immune therapy plus chemotherapy aimed at suppressing the cytokine storm associated with HLH. When a precipitating cause is identified, therapy is aimed at treating the underlying condition such as ATT for patients with TB-HLH, which may be sufficient in cases with mild presentation. Two treatment protocols have been developed in the treatment of primary HLH, HLH-94 [7] and HLH-2004 [16], which include agents such as dexamethasone, etoposide, and cyclosporine as back bones of therapy. Patients are initially placed on an induction regimen with these agents. Those who recover are weaned off therapy, while those who do not respond proceed to hematopoietic stem cell transplant. No clear guidelines exist on timing of initiation of immunomodulatory therapy in cases of secondary HLH.

Specifically, in the case of TB-HLH, literature review reveals that patients have been treated with ATT alone or ATT in combination with HLH-specific therapy. Patients who were started on ATT with or without immunotherapy had better outcomes than those who received no treatment at all [13]. Often, treatment with agents such as etoposide and cyclosporine is not feasible due to fulminant multi-organ failure at time of diagnosis, as was seen in our case. This further highlights the need for increased awareness of HLH, with paramount importance given to a search for secondary causes such as TB, as early diagnosis and prompt initiation of treatment could be lifesaving in an otherwise fatal illness.

## 4. Conclusion

TB-HLH is a life-threatening condition often with delay in diagnosis and a high fatality rate. A high index of suspicion is required by pathologists and clinicians as it may present with unusual findings such as spontaneous pneumomediastinum. Prompt treatment with ATT and potentially HLH-directed therapy may dramatically result in a better outcome.

## Figures and Tables

**Table 1:** HLH 2004 criteria [14].

Molecular diagnosis consistent with primary HLH (gene mutations)	Homozygosity or compound heterozygosity is required (*PRAF*1, *STX1*1, *RAB*27*A*, *STXBP*2, *UNC*13*D*, *SH*2*D*1*A, BIRC*4)
Below criteria are for secondary HLH (at least 5 of 8 criteria must be present)	
Fever	≥38.5 C
Splenomegaly	Spleen palpated >3 cm below the left costal margin
Hemophagocytosis	In bone marrow, spleen, lymph nodes, or liver
NK-cell activity	Low or absent according to local laboratory references
Soluble CD25	(i.e., soluble IL-2 receptor) ≥ 2,400 U/ml
Ferritin	≥500 *μ*g/L
Cytopenias (affecting ≥2 of 3 lineages)	Hemoglobin <9 g/dL
	Platelets <100 × 10^9^/L
	Neutrophils <1 × 10^9^/L
Hypertriglyceridemia and/or hypofibrinogenemia	Fasting triglycerides >265 mg/dL
	Fibrinogen <150 mg/dL

**Table 2:** H Score criteria [14].

Parameter	Status	Score
Known underlying immunosuppression	No	0
	Yes	18

Organomegaly	No	0
	Hepatomegaly or splenomegaly	+23
	Hepatomegaly and splenomegaly	+38

Number of cytopenias	1 lineage	0
	2 lineages	+24
	3 lineages	+34

Ferritin, ng/mL (or *μ*g/L)	<2000 = 0	0
	2000–6000	+35
	>6000	+50

Triglyceride, mg/dL (mmol/L)	<132.7 (<1.5)	0
	132.7–354 (1.5–4)	+44
	>354 (>4)	+64

Fibrinogen, mg/dL (g/L)	>250 (>2.5)	0
	<250 (<2.5)	+30

AST, U/L	<30	0
	>30	+19

Temperature, °F (°C)	<101.1 (38.4)	0
	101.1–102.9 (38.4–39.4)	+33
	>102.9 (>39.4)	+49

Hemophagocytosis in bone marrow aspirate	No	0
	Yes	+35
